# Multilocus variable-number tandem-repeat genotyping of Renibacterium salmoninarum, a bacterium causing bacterial kidney disease in salmonid fish

**DOI:** 10.1186/1471-2180-13-285

**Published:** 2013-12-06

**Authors:** Iveta Matejusova, Nicola Bain, Duncan J Colquhoun, Edward J Feil, Una McCarthy, Darryl McLennan, Michael Snow, David Verner-Jeffreys, I Stuart Wallace, Sarah J Weir, Malcolm Hall

**Affiliations:** 1 Marine Scotland Science, Victoria Road, P.O. Box 101, Aberdeen AB11 9DB, UK; 2 Norwegian Veterinary Institute, Pb 750, N-0106 Oslo, Norway; 3 Department and Biology and Biochemistry, University of Bath, Bath BA1 7AY, UK; 4 WA Fisheries and Marine Research Laboratories, Department of Fisheries, Government of Western Australia, PO Box 20, North Beach, 6020 Perth, Australia; 5 CEFAS, The Nothe, Barrack Road, Weymouth, Dorset DT4 8UB, UK

**Keywords:** Bacterial kidney disease, VNTR, Genotyping, *Renibacterium*, Salmonids

## Abstract

**Background:**

Bacterial kidney disease (BKD), caused by *Renibacterium salmoninarum*, is a bacterial disease of fish, which is both geographically widespread and difficult to control. Previously, application of various molecular typing methods has failed to reliably discriminate between *R. salmoninarum* isolates originating from different host species and geographic areas. The current study aimed to utilize multilocus variable number tandem repeats (VNTR) to investigate inter-strain variation of *R. salmoninarum* to establish whether host-specific populations exist in Atlantic salmon and rainbow trout respectively. Such information would be valuable in risk assessment of transmission of *R. salmoninarum* in a multispecies aquaculture environment.

**Results:**

The present analysis utilizing sixteen VNTRs distinguished 17 different haplotypes amongst 41 *R. salmoninarum* isolates originating from Atlantic salmon and rainbow trout in Scotland, Norway and the US. The VNTR typing system revealed two well supported groups of *R. salmoninarum* haplotypes. The first group included *R. salmoninarum* isolates originating from both Atlantic salmon and rainbow trout circulating in Scottish and Norwegian aquaculture, in addition to the type strain ATCC33209^T^ originating from Chinook salmon in North America. The second group comprised isolates found exclusively in Atlantic salmon, of mainly wild origin, including isolates NCIB1114 and NCIB1116 associated with the original Dee disease in Scotland.

**Conclusions:**

The present study confirmed that VNTR analysis can be successfully applied to discriminate *R. salmoninarum* strains. There was no clear distinction between isolates originating from Atlantic salmon and rainbow trout as several haplotypes in group 1 clustered together *R. salmoninarum* isolates from both species. These findings indicate a potential exchange of pathogens between Atlantic salmon and rainbow trout in Scottish and Norwegian aquaculture during the last 20 years. In a scenario of expansion of rainbow trout farming into the marine environment, appropriate biosecurity measures to minimize disease occurrence are advised. The present results also suggest that *R. salmoninarum* isolates circulating in European aquaculture over the last 20 years are genetically distant to the wild strains originally causing BKD in the rivers Dee and Spey.

## Background

*Renibacterium salmoninarum*[1] is a Gram-positive bacterium, belonging to the Micrococcus-Arthrobacter subgroup of the actinomycetes [2-4] and the causative agent of bacterial kidney disease (BKD), a chronic systemic disease of salmonid fish in both marine and freshwater environments [5]. Bacterial kidney disease was first reported in wild Atlantic salmon (*Salmo salar*) in the Rivers Dee and Spey (Scotland, United Kingdom) in 1930 [6,7] and similar disease signs were reported from North America in 1935 in brook trout (*Salvelinus fontinalis*), brown trout (*Salmo trutta*) and rainbow trout (*Oncorhynchus mykiss*) [8,9]. *Renibacterium salmoninarum* has an intracellular lifecycle and transmission, both horizontally through contact with infected fish/water or vertically inside fish ova, has been confirmed in many salmonid species [10-14]. Recent epidemiological studies have identified an association between the spread of BKD and anthropogenic activities [15,16].

Bacterial kidney disease is geographically widespread and has been reported from most countries where salmonid fish are cultured or naturally occurring. The disease is known to have the potential to cause high mortalities [17,18] and represents one of the most difficult bacterial diseases of fish to control due to its slow progression and lack of effective treatment. In Scotland, farmed Atlantic salmon and rainbow trout may be infected in both seawater and freshwater environments [19], although the contribution of wild fish to infection transmission is considered low [16].

Sensitive *R. salmoninarum* typing tools are required to improve BKD control through identification of sources of infection and transmission routes. Molecular typing methods, such as direct sequencing of the intergenic spacer (ITS1 and ITS) of ribosomal RNA and randomly amplified polymorphic DNA (RAPD) analysis [20-23], have shown a limited power to distinguish *R. salmoninarum* strains. Amplification of length polymorphisms in the tRNA intergenic spacer (tDNA-ILPs) has, however, offered improved discriminatory power with some potential for identification of *R. salmoninarum* isolates known to come from the same hatchery [23].

In Scotland, BKD and infection with *R. salmoninarum* are regulated under The Aquatic Animal Health (Scotland) Regulation 2009. From the available farm data, it appears that BKD persists longer on rainbow trout farms [24], compared with Atlantic salmon farms [16,19]. To date, all typing systems have failed to distinguish between *R. salmoninarum* strains originating from Atlantic salmon and rainbow trout [20,22,23], suggesting that individual isolates may represent a risk to both host species. Confirmation of this, applying a more sensitive typing tool, would be beneficial, for example, in a scenario of an expansion of rainbow trout sea water aquaculture. Application of appropriate biosecurity measures could then be applied to minimise risk of pathogen transmission.

In recent years, multilocus variable number tandem repeat analysis, based on amplification of short repetitive DNA sequences, has been found to be a rapid and simple typing technique that enables differentiation of bacterial strains displaying otherwise low genomic variation. The method has been used to discriminate between closely related strains of various human pathogenic microorganisms such as *Clostridium difficile*[25], *Bartonella henselae*[26], or *Streptococcus agalactiae*[27] as well as fish pathogenic species such as *Francisella noatunensis*[28]. The primary purpose of this study was therefore to investigate the genetic variation in *R. salmoninarum* isolated from Atlantic salmon and rainbow trout farms in Scotland using multilocus variable number tandem repeat analysis (VNTR). Additional samples from other countries were also included in the present study to put any observed variation into context and identify whether the present VNTR typing scheme can distinguish between *R. salmoninarum* collected from different geographic areas.

## Results

### Characterization of tandem repeat loci

In total, 32 tandem repeat loci were identified using either the Microorganisms Tandem Repeat Database or Tandem Repeats Finder (Additional file 1: Table S1). All loci were successfully amplified in 41 *R. salmoninarum* isolates (Additional file 2: Table S2) and sequences were analyzed for polymorphism (differences in number of tandem repeat units) (Accession numbers KF903677-KF904322). Sixteen of 32 studied loci were polymorphic (Table 1). The 16 monomorphic loci were excluded from the VNTR genotyping scheme.

**Table 1:** Number of alleles and variation in repeat span per polymorphic locus

Marker locus name*	**Number of alleles**	**Repeat number/span (bp)**
*BKD23*	4	3.7–6.7/33–60
BKD92	2	2.5–5.5/27–63
BKD143	5	9–14/37–57
*BKD305*	5	2.2–8.2/15–51
BKD396	2	2.6–4.6/16–32
BKD494	2	1.5–2.5/42–72
BKD526	2	1.5–2.5/42–72
BKD584	3	2.1–4.1/48–95
*BKD694*	3	1.9–2.9/19–27
BKD1023	2	1–2/37–71
*BKD1506*	3	5–8/34–56
BKD1850	2	4–5/43–55
*BKD1935*	3	1–3/52–154
*BKD2126*	8	5.3–11.3/37–79
BKD2770	4	2.8–4.8/26–44
BKD3038	2	2.6–4.6/46–81

*locus name reflects its position within genome of *R. salmoninarum* reference isolate ATCC33209^T^ (Accession number NC_010168). Loci in italics represent a minimum combined loci required to sufficiently recognized 17 *R. salmoninarum* haplotypes with the HGDI of value 0.81.

The allelic diversity ranged from two (BKD 92, 396, 494, 526, 1023, 1850 and 3038) to eight different alleles (BKD 2126) per locus. The largest observed variation in allele size was found in locus BKD2126 which varied between five to eleven repeats (Table 1). The VNTR typing system has a discriminatory power value of 0.81 and seventeen different haplotypes of *R. salmoninarum* were distinguished using 16 combined polymorphic VNTRs (Table 1, Table 2). A VNTR typing system relying on only six combined loci (BKD23, BKD305, BKD694, BKD1506, BKD1935, BKD2126) also sufficiently recognized 17 *R. salmoninarum* haplotypes, with the same discriminatory power value of 0.81.

**Table 2:** ***Renibacterium salmoninarum *** **isolates haplotype identified using multilocus tandem repeat sequencing**

**Haplotype**	**Isolate name**	**Country of origin**	**Host species**	**Environment (wild/farmed fish)**	**Data of isolation**
A	MT1470, MT1511^b^, MT2119^c^, MT2622^c^	Scotland	RT	FW, SW (F)	1994–2002
B	MT452^a^, MT839, MT1351, MT1363, MT1880, MT2979, MT3277^a^, MT3314, MT3315^b^, MT3402, N4245, N6642, N6552^d^, N6553^d^, N6694, N6765, N6863^e^, N6864^e^	Scotland, Norway	AS, RT	FW, SW (F)	1988–2009
C	MT2943, MT3320	Scotland	AS	SW (F)	2005–2008
D	MT3482, MT3483	Scotland	AS, RT	SW (F)	2009
E	N3769, N6695	Norway	AS, RT	FW, SW (F)	1997–2008
F	N5298	Norway	AS	SW (F)	2005
G	MT3106, MT3479, TERV	Scotland	AS, RT	FW, SW (F)	2006–2009
H	MT861	Scotland	AS	FW (F)	1990
I	MT1262	Scotland	AS	FW (F)	1992
J	ATCC33209	N. America	Chinook salmon	SW (F)	1974
K	MT3313	Scotland	RT	FW (F)	2008
L	MT444	Scotland	AS	SW (F)	1988
M	N5223	Norway	AS	SW (F)	2005
N	N6975	Norway	AS	SW (F)	2009
O	NCIMB1116	Scotland	AS	FW (W)	1960
P	NCIMB1114	Scotland	AS	FW (W)	1960
Q	N7443	Norway	AS	FW (W)	1985

^a,b,c,d,e^represent *R. salmoninarum* isolates from different disease outbreaks occurring on the same aquaculture site. RT – rainbow trout, AS – Atlantic salmon, FW – freshwater, SW – seawater, FA – farmed fish, W – wild wish.

### Phylogenetic relationships among *R. salmoninarum* isolates inferred from VNTRs

**Figure 1:**
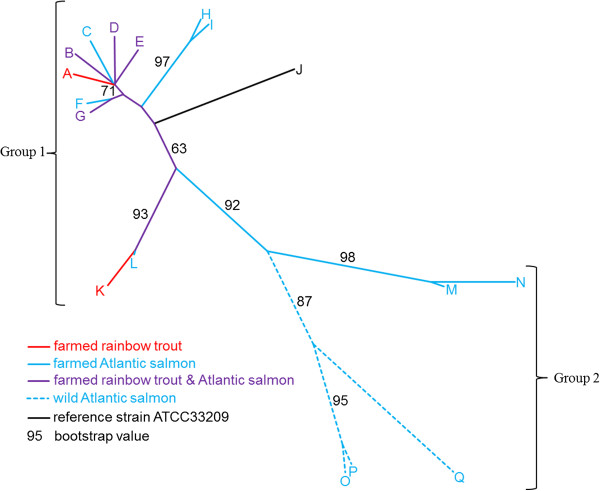
**Relationship among the observed haplotypes described in Table**2**.** Group 1 includes haplotypes A to L and Group 2 includes haplotypes M to Q. Bootstrap values indicate the level of support for clusters if higher than 50%.

The phylogenetic relationships among the *R. salmoninarum* strains inferred from 16 polymorphic VNTRs are illustrated in Figure 1. Two distinct groups comprising haplotypes A-L (group 1) and M-Q (group 2) were supported with a high bootstrap value (92%). Group 1 comprised *R. salmoninarum* from both Atlantic salmon and rainbow trout farmed in Scotland and Norway, recovered over a period of more than 40 years. This group also includes the type strain of *R. salmoninarum* ATCC33209^T^, recovered from a Chinook salmon (*Oncorhynchus tshawytscha*) in Oregon (USA) in the early 1970s. Strain B represented the most common haplotype, comprising 18 *R. salmoninarum* isolates from Atlantic salmon and rainbow trout farmed in Scotland and Norway over a period of more than 20 years. Strain B was one of five closely related strains (A, B, C, D, E) differing from each other at a single locus.

Group 2 represents *R. salmoninarum* isolates obtained uniquely from Atlantic salmon originating from Scotland and Norway. These isolates differed from group 1 at loci BKD396 and BKD1935. A moderately supported cluster within group 2, comprising strains O-Q, represented isolates exclusively from wild Atlantic salmon, including the Dee disease isolates NCIMB 1114 and 1116 associated with first occurrence of BKD in Scotland.

**Figure 2:**
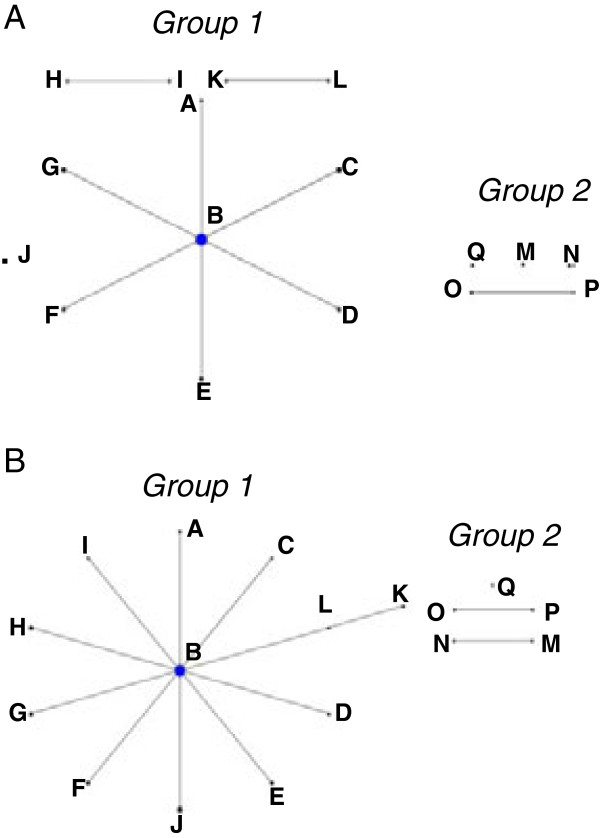
**eBURST diagram of *R. salmoninarum *** **population derived from the allelic variation in (A) 16 polymorphic loci or (B) 6 polymorphic loci.**

Similar clustering of *R. salmoninarum* isolates into two main groups was achieved using the eBURST algorithm based on either 16 or 6 polymorphic loci (Figure 2A,B). Using 16 polymorphic loci, a large radial cluster of 7 closely related haplotypes (A-G) was defined. Haplotype B was assigned as the most parsimonious “founder” of this group. Group 2 haplotypes occurred as a single pair O/P representing the Dee disease isolates and three singletons (L,M,N). Using eBURST, a loss in resolving power of the genotyping system was observed when the number of polymorphic loci included was reduced to 6 (Figure 2B).

The present VNTR typing scheme was also applied to investigate whether Scottish *R. salmoninarum* isolates can be distinguished from isolates originating from Norway. Within group 1, some association with country of origin was observed for haplotypes A, C, and G, uniquely obtained from Scottish aquaculture, while haplotype E represented *R. salmoninarum* from Norway. On the contrary, the most common haplotype B contained isolates obtained from aquaculture establishments in both countries.

## Discussion

The present study describes development and application of a VNTR typing system for *R. salmoninarum*, a bacterium affecting salmonid aquaculture worldwide and discusses the potential implications for disease management. In comparison to other genotyping methods used to study *R. salmoninarum* such as RAPD, tDNA-ILPs [20-23], multilocus VNTR typing offers a considerable improvement. Using a combination of sixteen VNTRs, 17 different haplotypes can be identified among 41 *R. salmoninarum* isolates. The discriminatory power of the present combined VNTR scheme was high, characterized by HGDI index of 0.81, indicating that two unrelated isolates will on 81% of occasions fall into different haplotypes. In contrast, no more than nine different tDNA-ILP profiles could be identified from a much larger collection of *R. salmoninarum* isolates [23]. In addition, VNTR represents a more reproducible typing system in comparison to techniques relying on random amplification under low-stringency parameters and accurate data from individual isolates can readily be shared between different laboratories. Although the discriminatory power of VNTR when applied to *R. salmoninarum* is lower than has been achieved with some human pathogenic bacteria such as *Bartonella* or *Streptococcus*[26,27], these later studies are based on significantly larger data sets usually gathered from wider geographic areas. If a larger *R. salmoninarum* data set becomes available in future, the VNTRs described in the present study should be applied to test its ability to trace disease outbreaks and connect individual infected farms with a source of infection.

The developed VNTR typing system separated the studied isolates into two well-supported groups. Group 1 clustered together 12 out of 17 *R. salmoninarum* haplotypes, including a wide range of isolates from Scotland, Norway and North America, from three different species of salmonid fish, spanning the period between 1974 and 2009. Several haplotypes of group 1 (B, D, E and G) comprised multiple isolates causing disease in both Atlantic salmon and rainbow trout, suggesting a relatively common historical transfer of the pathogen between these fish species. On the other hand, some association was found between rainbow trout and *R. salmoninarum* haplotype A and between Atlantic salmon and *R. salmoninarum* haplotypes C, F, H, I and L-Q. However, with the exception of haplotypes A and C, these haplotypes were represented by single isolations.

The present study concludes that using a data set of 41 isolates representing bacterium circulating in Scotland over a period of more than 20 years, there was no consistent division of *R. salmoninarum* isolates into two host specific populations. This result is consistent with the possibility that individual *R. salmoninarum* strains can infect both host species in environments where both species co-occur. The transfer of *R. salmoninarum* free stock to the marine environment could in theory eliminate disease transmission. However, the possibility that a carrier would be not detected, as a consequence of a potentially low infection prevalence and low diagnostic sensitivity of tests for asymptomatic stock, have to be considered [29]. The spatial separation of marine rainbow trout and Atlantic salmon farms into separate disease management areas in marine environment, as described in [16], can further reduce the risk of pathogen transfer between host species.

All previous *R. salmoninarum* typing systems have failed to reliably discriminate between European and US isolates [20,22,23]. This study identified the type strain ATCC33209^T^ originating from Oregon (US) as a unique strain (haplotype J) and its position within group 1 supports previous studies suggesting intercontinental spread of this pathogen related to anthropogenic activities such as movement of fish during expansion of the rainbow trout industry in Europe [30]. The present genotyping system could, however, clearly separate *R. salmoninarum* strains M-Q (group 2), indicating an origin not associated with ATCC33209^T^. The majority of these strains were of wild origin and have not been reported in wild or farmed fish since their original description in the 1930s. The locus BKD1935 was previously described by [22] referred to as the Exact Tandem Repeat A (ETR-A). It was demonstrated that the ETR-A can successfully separate the wild-fish isolates such as NCIMB1114 and NCIMB1116 (tandem repeat 1) from the farmed isolates such as MT452 and MT1363 (tandem repeat 2). Further investigation on a larger data set, focusing on loci BKD396 and BKD1935, which are solely responsible for differentiation between groups 1 and 2, might bring more insight into a relationship between farmed and wild *R. salmoninarum* strains and confirm the origin of *R. salmoninarum* in Scottish aquaculture.

## Conclusions

Cross-species infectivity of *R. salmoninarum* strains also has wider implications for marine ecosystems; including possible transfer of *R. salmoninarum* from farmed to wild fish or *vice versa*. In Scotland, recent studies provided evidence of a relatively low prevalence of *R. salmoninarum* in wild fish captured in close proximity to farms, suggesting that the transmission of this pathogen between wild and farmed fish is limited [16,31]. However, this scenario might not apply for other regions or countries such as England or Norway [32] and the described VNTR typing system can be utilized to identify and understand farmed and wild fish interactions in terms of *R. salmoninarum* transmission if a larger data set should become available.

## Methods

### Preparation of Renibacterium isolates and DNA extraction

Twenty-five *R. salmoninarum* isolates from confirmed disease outbreaks on Scottish farms were selected for this study. Number and selection of Scottish *R. salmoninarum* isolates represents the geographic range, habitat, frequency of disease outbreaks in the salmonid aquaculture sector, supply of fish stock and takes into account difficulties of bacteria culturing from asymptomatic fish and resuscitation of archived material. In addition, 14 Norwegian isolates and two isolates derived from the first successful cultivation of *R. salmoninarum* from the River Dee [7] were included. Isolate details including country of origin, date of isolation, host species and environment are summarized in Additional file 2: Table S2.

For Scottish strains, lyophilised cultures were resuscitated onto Mueller-Hinton L-cysteine agar (MHCA) containing polymyxin-B-sulphate, D-cycloserine, oxolinic acid and cycloheximide and incubated at 15°C for several weeks to allow growth. Suspensions of culture in 0.85% sterile saline were made to a turbidity of McFarland 1, serially diluted to 10^-4^ in sterile saline and 0.1 ml volume of the dilutions spread onto MHCA to achieve single colony purification. The Gram appearance and purity of individual colonies was confirmed before sub-culturing onto fresh MHCA and additional checks for purity and identity (API ZYM, ELISA using the Bios Chile kit) were carried out. At 4–6 weeks, each agar culture was scraped from the plate and suspended in sterile saline before pelleting at 2,400 × g. Genomic DNA was extracted from bacterial cultures using the MagAttract DNA mini M48 kit (Qiagen) and quantified using a ND-1000 Nanodrop Spectrophotometer (NanoDrop Technologies). For Norwegian strains, cryo-preserved isolates (−80°C) were resuscitated on kidney disease (KD) medium [33] followed by KD broth culture to an approximate turbidity of McFarland 1 prior to extraction of genomic DNA using the Gentra Puregene cell kit (Qiagen).

### Tandem repeat identification and amplification

The complete genome sequence of *R. salmoninarum* reference strain ATCC33209^T^[4] (Accession number NC_010168) was utilized to identify the repetitive DNA sequence regions using the Microorganisms Tandem Repeat Database (http://minisatellites.u-psud.fr) [34] and Tandem Repeats Finder (TRF version 4.03) (http://tandem.bu.edu) [35]. Tandem repeats with at least two repeat units per locus and a repeat unit length of between 4 and 80 bp were selected for further analysis.

Primers for amplification of each locus were designed using OligoPerfect™ Designer (http://tool.invitrogen.com) and their specificity tested using BLAST (blastn) searches. Loci were amplified using the primer pairs listed in Additional file 1: Table S1. Each reaction consisted of 1 × PCR buffer (Bioline), 1.5 mM MgCl_2_, 200 μM dNTPs, 10 μM of each primer, 1 U BioTaq (Bioline) in a final volume of 20 μl. The cycling conditions were 35 cycles of: 95°C for 1 min, 50 or 55°C (see Additional file 1: Table S1) for 1 min, 72°C for 1 min, followed by a final elongation step of 72°C for 5 min. Amplified products were visualized on a 1% ethidium bromide-stained agarose gel (Invitrogen) and purified using ExoSAP IT or ExoStar 1-Step (GE Healthcare). Approximately 15 ng of purified PCR product was sequenced, utilising the same primers as in the amplification reaction using the GenomeLab DTCS Quick Start kit (Beckman Coulter) and the automated CEQ8800 DNA Sequencer (Beckman Coulter).

### Tandem repeat analysis

Each type (size) of repeat, identified by sequencing, at each locus was assigned a unique allele identifier. Data were imported from a Microsoft Office Excel 2003 generated comma-separated-value data file and analysed using version 2.14.0 of the R statistical computing environment [36]. The permutations of alleles across 16 polymorphic loci were used to define distinct haplotypes.

Gross differences between haplotypes (identified using 16 polymorphic loci) were measured with the Hamming distance [37] and used to construct an unrooted neighbor-joining tree using version 1.6-0 of the supplementary R package phangorn [38]. To simplify interpretation of results, haplotypes were named A-Q on the basis of their respective position in the phylogenetic tree. Support for clusters was evaluated using the bootstrap test of phylogeny (1000 repeats) and clusters with values of less than 50% collapsed [39]. The clustering of very closely related haplotypes, defined as those differing at only one locus, was examined using eBURST v 3.0 [40]. Homoplasy and extent of recombination events were investigated using Splits Decomposition, as implemented in Splitstree v 4 [41], by depicting conflicting signals in the data caused by recombination events. The resulting network was consistent with the phylogenetic analysis, and no reticulation was evident, indicating that the evolutionary relationships have not been affected by recombination or homoplasy (data not shown).

The discriminatory power of a typing system was estimated using the Hunter-Gaston discriminatory index HGDI [42]. The index provides a probability that two randomly sampled unrelated isolates will be placed into different typing groups/haplotypes. The minimum number of loci required to distinguish all the strains was determined.

## Competing interests

The authors declare that they have no competing interests.

## Authors’ contributions

All authors contributed to the study design. IM, NB, DM, and SJW contributed to molecular studies. UM and DJC prepared bacterial cultures. IM, EJF and MH analysed the molecular data. IM wrote the manuscript and BN, DJC, EJF, UM, DJV and MH revised the manuscript. All authors read and approved the final manuscript.

## Supplementary Material

Addition al file 1: Table S1 List of amplified and analysed tandem repeat loci within the *R. salmoninarum* genome. Click here for file

Additional file 2: Table S2 List of *R. salmoninarum* isolates used for tandem repeat polymorphism analysis. Click here for file
