# Supplementary material for: Multilocus variable-number tandem-repeat genotyping of Renibacterium salmoninarum, a bacterium causing bacterial kidney disease in salmonid fish
Source: BMC Microbiol. 2013 Dec 6;13:285. doi: 10.1186/1471-2180-13-285 (PMC4029610; doi:10.1186/1471-2180-13-285)
Supplement: Addition al file 1: Table S1 — List of amplified and analysed tandem repeat loci within the R. salmoninarum genome. [file 1471-2180-13-285-S1.doc]

**Table 1S** List of amplified and analysed tandem repeat loci within the *R. salmoninarum* genome

* loci found in The Microorganisms Tandem Repeat Database, + loci found using Tandem Repeats Finder Program. Primer sets in bold used an annealing temperature of 50 °C, primer sets in normal font used an annealing temperature of 55 °C in PCR reaction. Underlined loci were selected for VNTR scheme, ** copy number refers to the reference isolate ATCC33209^T^ as indicated by the software used

| Loci position (bp) | Tandem number** | | Sequence (consensus pattern) | Primer set sequence (5'-3') | Putative function of ORF |
| --- | --- | --- | --- | --- | --- |
| 23000* | 5.7 | | AATTGCAGC | **23F-CGGAACTGATCCATAGTGAC**  **23R-CAATTGACCAGATTGACGTA** | esterase |
| 92000+ | 2.5 | | GGTGAACGTTTT | 92F-TCGTTGACAGCTCATACCA  92R-CGGCCAAATATCTCGGTAAA | hypothetical membrane protein |
| 143000+ | 9 | | TGGC | 143F-CTGGGCGATCTGATCTCATT  143R-GGCTAGCATCCAGATCGAAA | disaccharide transport system permease protein |
| 305000+ | 7.2 | | CCAGGT | 305F-GGTCGTCAACGAATCCAAGT  305R-GCTTCGCTGCCAGTACGAC | inositol 2-dehydrogenase |
| 364000* | 2.3 | | TGTTTTTTGAGAACTCAATAGTGTGTTT | **364F-CAAAACCTTACGATTTGGAG**  **364R-TTCGACTTGCATGTGTTAAT** | 16S ribosomal RNA gene |
| 396000+ | 4.6 | | CAGCATG | 396F-GCAGCTGGACGATTTCTTTC  396R-GACGGTTTCGCCTAGCTTC | formamidopyrimidine-DNA glycosylase |
| 460000* | 2.2 | | GAACAAGACTTTCTGCTCAGAAACCG | 460F-GGATGAGTTCTTCTTCATGG  460R-CCTTAAGTACTCGGTGTTGG | acetyl/propionyl CoA carboxylase |
| 494000* | 2.5 | | GAGCTGAAACGGTTACGGCGTGAGAACGCT | 494F-GTATCCGCAGGAGTTGAAG  494R-GGTGAGATCGTGTTTTATCC | sorbitol operon regulator |
| 526000* | 2.5 | | CCGTTTCAGCTCAGCGTTCTCACGCCGTAA | **526F-ACCGCAATCCATTATTCTC**  **526R-CGGAGACTTTCTTGTTTGAG** | hypothetical protein |
| 543000+ | 2.6 | | CGGGTGCTGGTTT | 543F-CGAAGTCTTTGGCTTTTTCG  543R-GATCCAGAAATTGCCCAGAA | hypothetical membrane protein |
| 546000* | 4.6 | | TCGGGTGGCCCTCGTCCAGCAGCTGGT | **546F-GCCACTTCGGAAGGTATG**  **546R-CCTTGCCAAAGCCACCAC** | translation initiation factor IF-2 |
| 584000* | 3.1 | | TACGCCGCCGAGCTCTCCACCGAC | 584F-CTCAGTGATCAGGACAAGAAG  584R-CTCAATGGATCCAAACTCTG | dihydrodipicolinate synthase |
| 674000* | 2.3 | | TGTTTTTTGAGAACTCAATAGTGTGTTT | **674F-ATTACGGCCAAAACTAGATG**  **674R-TTCGACTTGCATGTGTTAAG** | hypothetical protein |
| 694000+ | 2.9 | | GTGCTGCCT | 694F-AACTCTGGGCAAATCACCAC  694R-TTGCCAGCCATATTCAGTTG | NUDIX hydrolase |
| 702000* | 1.9 | | TTTTGTCCCCGCCAGCAGCGCTACGCGGGGACATAAACAC | **702F-CTGTGCAACGTCTGGTAAG**  **702R-GTGTTAATGTTCCGGGTTTA** | transcriptional regulator, GntR family |
| 1023000* | 2 | | GGCCAGGCCCACCGCGGCTCGGCCGGGTTTCGTG | **1023F-GGTCGGTAGCTTCAGTGAT**  **1023R-CAGACATTGCCCAGTTGA** | thiamin-phosphate pyrophosphorylase |
| 1419000* | 3.7 | | CAGCCAGAGCCATAT | 1419F-GCTGGAATCTTTAGAACGTG  1419R-CGTTTGGTATCTAAGCTCAAG | MoxR-like ATPase |
| 1506000+ | | 7 | GCAACCA | 1506F-AGTGACGGGTTCACAGCATT  1506R-CGACAGCTCAGCGATAACAA | hypothetical protein |
| 1541000* | | 2.1 | TTGCGCAGAGTTCTGCGCGGTACC | 1541F-AAGTCCTCGACCCCTAGC  1541R-AGAACATGCTGCTAATAGGC | formamidopyrimidine-DNA glycosylase |
| 1850000* | | 5 | GACCGTGCAAG | **1850F-TTCCGACTATTTCGGTTTAG**  **1850R-CGGTGACAATAACCTGATTT** | aminopeptidase N |
| 1935000* | | 2.1 | CTAAGTCCGGCGCTGCGGCGACGGCAATCCGGGCCGTGGCTACGGCGTCGT | 1935F-CACCGAAGTCAGATAAGAGG  1935R-TAGGCGTAGTGCTCTATGGT | hypothetical protein |
| 2003000+ | | 2.7 | GCCACAGCGA | 2003F-ATCCGTCAGGGCAGTATCAC  2003R-TGAACGCTCAACAATTCTGC | hypothetic protein |
| 2086000* | | 3.1 | CCCATTGCTCTGCCAAG | **2086F-AGCTGTTCAAAATGAGCTTG**  **2086R-GGCTATTTCGTTGCAGTTAG** | ribulose-phosphate 3-epimerase |
| 2124000+ | | 3.1 | AGCACCCATT | 2124F-CAGACGCCCAAGACGATATT  2124R-GCAAAACGATGACTGGGAAT | cation-transporting ATPase |
| 2126000+ | | 6.3 | GCTTAGG | 2126F-TTGCGATGCTGAACTCATTC  2126R-GCGAAAGTTCCAGAAAGCTC | HNH endonuclease domain protein |
| 2189000+ | | 2.8 | CATTGGTGG | 2189F-CGAAGTATGGCTCGGAGAAG  2189R-TGCGCTGTACCTGTATGTCG | glutamine transport permease protein |
| 2356000* | | 4.3 | CCGGTCGTGGTGCCG | **2356F-AAACTGGAGCTGATGGTCTA**  **2356R-AAGTACGGATCGCTAAACAA** | Xaa-Pro dipeptidyl-peptidase |
| 2728000+ | | 2.6 | GCGGGTAGAT | 2728F-CCATATCCAGGTGCGAGACT  2728R-CCTCAGATGCCCTAATCCAG | aldose 1-epimerase |
| 2763000* | | 2.4 | CCGCCACCGGTAGTTCCGCCAGTCAAC | **2763F-GTCAACCCTTGAAGACCAG**  **2763R-AATGTTGACACCGACTGAAC** | cell wall endopeptidase |
| 2770000+ | | 3.8 | TCTCGGCGA | 2770F-GGAGTCCTTACCGGTCTTCC  2770R-CCTTTGCCAATGAACCAAAC | hypothetical protein |
| 3038000+ | | 2.6 | AAAGCCAGCGCTTCGGG | 3038F-TGGGTGTACCAAACCTGATG  3038R-GAAACCACTAGGGACCGTCA | D-mannonate oxidoreductase |
| 3117000* | | 1.9 | CGGCTCAATCAAGATCAACTTGGCTGA | 3117F-GTAGGCGACATTGTGAAGAC  3117R-GGTTGTTCAGAACCTGAGTC |  |
