# Supplementary material for: Multilocus variable-number tandem-repeat genotyping of Renibacterium salmoninarum, a bacterium causing bacterial kidney disease in salmonid fish
Source: BMC Microbiol. 2013 Dec 6;13:285. doi: 10.1186/1471-2180-13-285 (PMC4029610; doi:10.1186/1471-2180-13-285)
Supplement: Additional file 2: Table S2 — List of R. salmoninarum isolates used for tandem repeat polymorphism analysis. [file 1471-2180-13-285-S2.doc]

**Table 2S** List of *R. salmoninarum* isolates used for tandem repeat polymorphism analysis. RT – rainbow trout, SA – Atlantic salmon, CHS – Chinook salmon, FW – freshwater, SW – seawater. ^a,b,c,d^ and ^e^ represent *R. salmoninarum* isolates from different disease outbreaks occurring on the same aquaculture site. ** Data not available

| Isolate | Host | Environment | Isolation date | Geographic region |
| --- | --- | --- | --- | --- |
| NCIMB1114 | SA | FW | 1960 | UK, Grampian |
| NCIMB1116 | SA | FW | 1960 | UK, Grampian |
| MT444 | SA | SW | 1988 | UK, Western Isles |
| MT452 ^a^ | RT | FW | 1988 | UK, Dumfries and Galloway |
| MT839 | SA | SW | 1990 | UK, Highlands |
| MT861 | SA | FW | 1990 | UK, Highlands |
| MT1262 | SA | FW | 1992 | UK, Highlands |
| MT1351 | SA | SW | 1993 | UK, Highlands |
| MT1363 | RT | SW | 1993 | UK, Strathclyde |
| MT1470 | RT | FW | 1994 | UK, Tayside |
| MT1511^b^ | RT | FW | 1994 | UK, Strathclyde |
| MT1880 | SA | SW | 1996 | UK, Strathclyde |
| MT2119^c^ | RT | SW | 1998 | UK, Strathclyde |
| MT2622 ^c^ | RT | SW | 2002 | UK, Strathclyde |
| MT2943 | SA | SW | 2005 | UK, Highlands |
| MT2979 | RT | FW | 2005 | UK, Highlands |
| MT3106 | RT | FW | 2006 | UK, Strathclyde |
| MT3277 ^a^ | RT | FW | 2008 | UK, Dumfries and Galloway |
| MT3313 | RT | FW | 2008 | UK, Central |
| MT3314 | SA | SW | 2008 | UK, Shetland |
| MT3315 ^b^ | RT | FW | 2008 | UK, Strathclyde |
| MT3320 | SA | SW | 2008 | UK, Shetland |
| MT3402 | SA | SW | 2009 | UK, Shetland |
| MT3479 | SA | SW | 2009 | UK, Orkney |
| MT3482 | RT | SW | 2009 | UK, Strathclyde |
| MT3483 | SA | SW | 2009 | UK, Strathclyde |
| TERV | RT | FW | 2009 | UK, Strathclyde |
| N3769 | SA | FW | 1997 | Norway |
| N4245 | SA | FW | 2001 | Norway |
| N5223 | SA | SW | 2005 | Norway |
| N5298 | SA | SW | 2005 | Norway |
| N6642 | SA | SW | 2008 | Norway |
| N6552^d^ | SA | SW | 2008 | Norway |
| N6553 ^d^ | SA | SW | 2008 | Norway |
| N6694 | RT | SW | 2008 | Norway |
| N6695 | RT | SW | 2008 | Norway |
| N6765 | RT | SW | 2009 | Norway |
| N6863^e^ | RT | SW | 2009 | Norway |
| N6864 ^e^ | RT | SW | 2009 | Norway |
| N6975 | SA | SW | 2009 | Norway |
| N7443 | SA | FW | 1985 | Norway |
| ATCC33209 | CHS | SW | 1974 | United States |
